# Evaluating mobile harm reduction services for youth and young adults

**DOI:** 10.3389/fpubh.2024.1375323

**Published:** 2024-05-22

**Authors:** Ellis J. Yeo, Elizabeth Hausman, Elizabeth Noyes, Avik Chatterjee

**Affiliations:** ^1^Harvard Medical School, Boston, MA, United States; ^2^College of Social Work, University of Kentucky, Lexington, KY, United States; ^3^Department of Otolaryngology-Head and Neck Surgery, Mass Eye and Ear, Boston, MA, United States; ^4^Department of Medicine, Boston University School of Medicine, Boston, MA, United States; ^5^Boston Health Care for the Homeless Program, Boston, MA, United States

**Keywords:** harm reduction, youth, mobile health, substance use disorder, addiction

## Abstract

The adolescent and young adult (AYA) population has experienced an increase in both emergency room visits and deaths related to substance use. However, AYA are less likely to engage in existing addiction treatment infrastructure. A youth-specific mobile harm reduction program has the potential to reduce substance-related morbidity and mortality including infections, overdose, and death. Launched in 2019, the Community Care in Reach AYA pilot program seeks to address the difference in patterns of substance use between AYA and adults. The results of this evaluation suggest the importance of a youth-oriented program in increasing AYA engagement with harm reduction.

## 1 Introduction

The adolescent and young adult (AYA) population has experienced an increase in both emergency room visits and deaths related to opioid use from the 1990s to 2010s (1, 2). Harm reduction practices and medication for opioid use disorder (MOUD) have the potential to reduce drug-related morbidity and mortality including infections, overdose, and death. AYA are less likely to engage in substance use disorder (SUD) treatment, and though they can benefit from harm reduction services, they engage differently with these services compared to adults (3). Specifically, AYA are less likely to access harm reduction resources and may rely on social networks to reduce risk, such as using substances in the presence of others (4). Presence of older participants, fear of law enforcement, and age restrictions may be a barrier to harm reduction access (5). Therefore, harm reduction services must be tailored to the unique needs of AYA (6). AYA are a unique, difficult-to-reach population within addiction medicine and describe numerous barriers to engaging in harm reduction services, including lack of youth-tailored services, fear of law enforcement, stigma, lack of outreach, and lack of perceived need for services (5). There is minimal existing research on how to best provide substance use care and harm reduction services to this population.

## 2 Context

To address the evolving needs of people who use substances, Community Care in Reach (CCIR; formerly CareZone), launched a mobile initiative in Boston in 2018 in partnership with numerous community organizations including Boston Health Care for the Homeless Program (BHCHP) and Boston Public Health Commission’s AHOPE program to provide services (7, 8). This initiative focused on mobile harm reduction outreach to all people who use substances, including distribution of naloxone and unused syringes, as well as offering MOUD, particularly buprenorphine. After a successful year of the CCIR van’s initial Boston pilot, the Kraft Foundation and the Grayken Center at Boston Medical Center partnered with the Family Team at BHCHP (which is an interprofessional team that has been providing clinical services to homeless families and AYA in Boston since 1985) and Bridge Over Troubled Water (which provides emergency shelter and social and clinical services to AYA in Boston) to develop a pilot program to utilize CCIR to reach AYA (9).

In the summer of 2019, we conducted a needs assessment to help inform implementation of the CCIR AYA pilot. This revealed that while substance use was common, patterns of substance use were very different among AYA compared to adults, in terms of the substances they commonly use and the risk perception of their substance use, suggesting a need for services tailored to their unique needs (10). AYA were not familiar with the term “harm reduction” and did not have consistent ways to access harm reduction programs, substance use disorder treatment, and other health services (11). Developed with these findings in mind, the CCIR AYA pilot began in the fall of 2019.

This program demonstrates tremendous potential to provide low-threshold, on-demand harm reduction services tailored to young people and thereby reduce drug-related harms among this population. The purpose of this research study was to assess the impact, uptake, and acceptability of services as well as to determine gaps in care and areas for improvement. We conducted a mixed methods evaluation of CCIR’s AYA mobile harm reduction pilot program through perspectives of AYA and CCIR providers including a chart review of patient encounters, participant surveys, and patient and provider interviews.

## 3 Programmatic elements

The CCIR AYA pilot was staffed by a physician or nurse practitioner with experience working with AYA in addiction medicine, two outreach members: a harm reduction specialist and a peer recovery specialist, both of whom had prior experience working with AYA and matched their age range. The van is a 24′ mobile medical unit large enough for a private, fully-equipped exam room, a sink, a wheelchair lift, a small reception area, and two refrigerators for vaccine and food storage (see Supplementary Figures). Services available on the van included medications for substance use disorders (particularly, sublingual buprenorphine—long-acting injectable buprenorphine was not available due to storage regulations), STI testing, basic primary care, distribution of harm reduction supplies (safe sex kit, safer substance use kit, unused syringes, naloxone), and case management. In a typical week, the van conducts outreach and delivery services twice weekly, most commonly Tuesdays and Thursdays from 4–8 pm. The van drives to areas where AYA who use substances commonly gather to conduct outreach and offer services, such as skate parks, community parks, and near established youth organizations such as BOTW.

As with other clinical services in Boston, the COVID-19 pandemic put a three month pause on CCIR AYA outreach. After the pause, providers reflected on facilitators and barriers to the program prior to COVID and considered how COVID changed patterns of where AYA spent time and how they accessed services. BHCHP re-launched the AYA pilot with several changes in place, seeking to update where and how they provided services. Most notably, social media was integrated in order to help connect with AYA, advertise CCIR outreach timing and location, distribute educational material, and coordinate delivery of harm reduction supplies.

## 4 Evaluation

### 4.1 Methods

The purpose of this research study was to understand the impact and acceptability of harm reduction services currently being offered to adolescents and young adults by the CCIR van. To gain a full understanding of the CCIR van service delivery and impact, we gathered information from both AYA participants who utilize the CCIR van’s services and providers who work on the CCIR van.

#### 4.1.1 Chart reviews

We conducted a chart review of clinical records of AYA patients who were seen by the CCIR van. From the patient charts, we gathered information including age, gender, language, medications prescribed, chief complaints, comorbidities, medical history, and purpose of clinical encounter.

#### 4.1.2 Surveys

We conducted surveys to assess participants’ satisfaction with CCIR van services. We surveyed participants who were 18–24 years of age and spoke English. Surveys lasted approximately 15 min. A study staff member accompanied van outreach staff on their outreach rounds to reach out to AYA. Study staff and outreach workers approached youth who attended the services to describe the survey to potential study participants and provide fact sheets. There was no requirement to have received services from the van in order to fill out the survey. The study was administered online through a QR code link. Participants had the option to complete the survey on their own or with the help of study staff. Participants were also recruited through various recruitment posts on Instagram. After completing the survey, participants received a $15 gift card to CVS.

#### 4.1.3 Interviews

We also recruited patient and provider participants for qualitative interviews to learn more about their engagement in services and/or views regarding the CCIR van. The data was de-identified but there may have been overlap in patient participants with the survey respondents. The semi-structured interviews lasted approximately 30 min. Interviews occurred in private spaces, such as inside the CCIR van or by Zoom. Participants received a $25 gift card after participation.

Study staff ensured informed consent and understanding of the study from participants before beginning the interviews. Interviews were audio recorded and participants were informed of and consented to this. Audio recordings were taken via encrypted Zoom recordings and stored on secure BMC-approved online storage site. All interviews were transcribed. A third party transcription company performed transcription and two authors conducted thematic analysis to determine themes that arose from the interview data.

### 4.2 Outreach visits

From December 23, 2019, through March 18, 2020, the CCIR mobile van made 284 contacts and distributed 520 syringes and 421 units of naloxone. From June 15 through October 19, 2020, the mobile van made 593 contacts and distributed 248 units of naloxone and 590 syringes during 32 visits.

#### 4.2.1 Chart review findings

We reviewed 35 total clinical encounters with a physician or nurse practitioner on the CCIR mobile van from November 2019 to April 2022. Of these encounters, one patient was under 18 years of age and seven patients were over the age of 24 at the time of the encounter, leaving 17 patients with 27 encounters where patients were between 18 and 24 years of age.

Patients had a mean age of 22.2 years, and 64.7% identified as male and 5.9% identified as non-binary or genderqueer. Patients predominantly identified as Black (41.2%), white (35.3%), and Asian/Pacific Islander (11.8%). Seven patients (41.2%) identified as Hispanic.

Patient’s past medical history as listed in their clinical charts contained a variety of comorbid diagnoses. Skin conditions were most common among participants (52.9%), followed by high BMI or obesity (35.3%), chronic pain (29.4%), and STIs (23.5%). All participants (100%) had undergone STI testing in the past and all but one (94.1%) had undergone HIV and Hepatitis C testing. Of those with available results, no patients were found to have HIV or Hepatitis C. The majority of patients (88.2%) had at least one psychiatric diagnosis, most commonly major depressive disorder (64.7%), post-traumatic stress disorder (41.2%), and attention deficit-hyperactivity disorder (41.2%).

The majority of patients had at least one diagnosed substance use disorder (82.4%). Tobacco use disorder or cigarette nicotine dependence (41.2%) was most common, followed by alcohol use disorder (35.3%), cannabis use disorder (17.6%), and opioid use disorder (11.8%). Tobacco (76.5%), alcohol (64.7%), and marijuana (58.8%) were by far the most reported substances used. Only two patients reported using opioids in their lifetime (11.8%). One patient had a documented history of overdose. Eight patients (47.1%) had received prior treatment for substance use disorders. Most patients (70.6%) were connected to other harm reduction services, including the BHCHP Family Team and flagship clinic (47.1%), and Bridge over Troubled Waters (41.2%). Most patients (82.4%) had at least one prior visit with a BHCHP Family Team provider before attending the CCIR van, and the majority (76.5%) also had a subsequent visit with a BHCHP Family Team provider.

The main issues addressed at the encounters (*n* = 27) were STIs and STI testing (55.6%), acute or subacute injuries (25.9%), substance use (18.5%), chronic medical problems (14.8%), and mental health (11.1%). Three (11.1%) patients were prescribed treatment for a substance use disorder during the encounter: one (3.7%) was prescribed nicotine gum, one (3.7%) was prescribed buprenorphine, and one (3.7%) was given a referral for naltrexone injections at the BHCHP clinic.

### 4.3 Survey findings

Fourteen AYA between the age of 18 and 24 years of age completed the survey in its entirety during the study period. Nearly all participants reported having a cellphone (92.9%) and reported this as their primary method of accessing the internet (92.9%). Instagram, Snapchat, and email were the most common means of accessing the internet (57.1%) (Table 1).

**Table 1:** Demographics and social media usage of 14 adolescent and young adult survey respondents who engage in mobile harm reduction and addiction services.

	*n*	%
Gender identity	14	
Female	7	50%
Male	7	50%
Transgender	0	0.0%
Sexual orientation		
Straight	12	85.7%
Bisexual	1	7.1%
Gay	1	7.1%
Race		
American Indian or Alaska native	1	7.1%
Black or African American	6	42.9%
White	8	57.1%
Ethnicity		
Spanish, Hispanic, or Latinx	6	42.9%
Housing Status		
An apartment/house that the participant rents or owns	4	28.6%
On the street or in another outdoor place (like a park)	2	14.3%
Drop-in center or emergency shelter	2	14.3%
Transitional housing program	1	7.1%
A parent/family member’s house/apartment	1	7.1%
Do not know/do not want to respond	4	28.6%
Do you have a cellphone?		
Yes	13	92.9%
What device do you generally use to access the internet?		
Cell phone	13	92.9%
Personal computer	2	14.3%
Public computer	2	14.3%
How do you generally access the Internet?		
Instagram	8	57.1%
Email	8	57.1%
Web browser	6	42.9%
Snapchat	8	57.1%
Apps	5	35.7%
Facebook	7	50.0%
Text message	7	50.0%

Eight participants (57.1%) reported using alcohol in the past week, eight used marijuana (57.1%), one (7.1%) used cocaine and ecstasy. None reported opioid use in the past week. Three participants (21.4%) reported using opioid pills a couple times over the past year and no participants reported heroin or fentanyl use. Smoking was the most common means of using drugs (71.4%); participants also reported snorting (14.3%) and swallowing (21.4%). No participants reported injection as their most common means of using drugs (Tables 2, 3).

**Table 2:** Harm reduction and substance use practices reported by 14 adolescent and young adult who engage in mobile harm reduction and addiction services.

	*n*	%
Have you heard the term “harm reduction” before?		
Yes	7	50.0%
No	6	42.9%
Do not know or unsure	1	7.1%
Where did you hear about the CCIR mobile van?		
Word of mouth	4	28.6%
Friend/peer/acquaintance	2	14.3%
I have never heard of the CCIR van	4	28.6%
Other	4	28.6%
In the past year, were you prescribed any of the following medications for opioid use disorder?		
Methadone	1	7.1%
I wasn’t prescribed any medications for opioid use disorder	11	78.6%
Prefer not to say	2	14.3%
Have you ever heard of Narcan, a medication also known as Naloxone?		
Yes	9	64.3%
No	5	35.7%
Do you know where you can buy or obtain Naloxone/Narcan?		
Yes	6	42.9%
No	8	57.1%
Do you know how to use Narcan/Naloxone?		
Yes	7	50.0%
No	7	50.0%
Do you carry a Naloxone/Narcan kit with you most of the time?		
Occasionally	2	14.3%
No	10	71.4%
Yes	2	
Do you know how to use fentanyl test strips?		
Yes	5	35.7%
No	9	64.3%
If your drugs tested positive for fentanyl (before you use), would you change the amount you use?		
Yes, would use less	5	35.7%
No, nothing would change	6	42.9%
Prefer not to say	3	21.4%
Are you connected to health care services anywhere?		
Yes	9	64.3%
No	3	21.4%
Do not know or do not want to respond	2	14.3%
Are you connected to mental health services, like a psychiatrist or therapist, anywhere?		
Yes	7	50.0%
No	4	28.6%
Do not know or do not want to respond	3	21.4%
In the past week, which of the following substances have you used? (check all that apply)		
Alcohol (beer, liquor, wine)	8	57.1%
Marijuana	8	57.1%
Opioids (e.g., heroin, fentanyl, oxycodone, morphine, dilaudid, suboxone)	0	0.0%
Cocaine (powder or crack)	1	7.1%
Ecstasy/MDMA/Molly	1	7.1%
Other*	5	35.7%
How do you generally use drugs? (Select all that apply)		
Smoking/inhalation	10	71.4%
Snorting	2	14.3%
Injecting	0	0.0%
Swallowing	3	21.4%
Other	1	7.1%
In the past year, how often did you inject?		
Once or a couple of times	1	7.1%
Never	11	78.6%
Do not know or do not want to respond	2	14.3%
How often do you use drugs alone?		
Never	6	42.9%
Occasionally	1	7.1%
Often	2	14.3%
Always	2	14.3%
Prefer not to say	3	21.4%
What are some of the reasons YOU use drugs alone?		
It’s safer to be alone	4	28.6%
It’s more convenient and comfortable to use alone	3	21.4%
I do not want to share	1	7.1%
I do not want others to know that I’m using drugs	1	7.1%
I do not have anyone else to use with	2	14.3%
I never use alone	5	35.7%
Have you ever overdosed?		
Yes**	1	7.1%
No	12	85.7%
Do not know or do not want to respond	1	7.1%
In the past year, have you SEEN an accidental overdose in someone using any opioids?		
Yes	6	42.9%
No	7	50.0%
Prefer not to say	1	7.1%
Did you give Naloxone/Narcan to the person that overdosed during the last opioid overdose you witnessed? (*N* = 6)		
Yes	2	33%
No***	4	66%
Have you heard about the Good Samaritan Drug Overdose Act?		
Yes	3	21.4%
No	10	71.4%
Prefer not to say	1	7.1%

*Under ‘other’, survey participants reported using spice and mushrooms. Two participants wrote ‘none’. **Survey participant indicated that only Opioids (e.g., heroin, fentanyl, oxycodone, morphine, dilaudid, suboxone) contributed to their overdose. ***Survey participant indicated that they did not know how to use Naloxone/Narcan.

A total of five participants (35.7%) reported having used drugs alone, noting reasons such as it is safer to be alone (28.6%), more convenient and comfortable to use alone (21.6%), and not having others to use with (14.3%). One participant (14.3%) reported having an opioid-related overdose. Six participants (42.9%) reported seeing someone else accidentally overdose with opioids. Two (33%) reported administering Naloxone to the last person they witnessed overdose. Only three participants (21.4%) reported that they had heard of the Good Samaritan Drug Overdose Act (Table 2).

Half of the respondents had heard of the term “harm reduction” before. Most participants (64.3%) had heard of Naloxone previously, though only 42.9% reported knowing where to obtain it and half reported knowing how to use it. Under half participants (42.9%) had heard of fentanyl test strips before, and five participants (35.7%) reported knowing how to use fentanyl test strips. Five participants (35.7%) reported they would use less if their drugs tested positive for fentanyl, and six (42.9%) reported they would not change their use patterns (Table 2).

**Table 3:** Substance use frequency in the past year reported by 14 adolescent and young adult who engage in mobile harm reduction and addiction services.

In the past year, how often have you used…	Every day	Once or a couple of times	Never	Do not know or do not want to respond
Alcohol (beer, liquor, wine)	1 (7.1%)	9 (64.3%)	2 (14.3%)	2 (14.3%)
Marijuana	3 (21.4%)	8 (57.1%)	1 (7.1%)	2 (14.3%)
Heroin/fentanyl	0 (0%)	0 (0%)	12 (85.7%)	2 (14.3%)
Opioid pills (oxycodone, oxycontin, Percocet, vicodin, dilaudid, hydrocodone)	1 (7.1%)	2 (14.3%)	10 (71.4%)	1 (7.1%)
Cocaine (powder or crack)	0 (0%)	2 (14.3%)	10 (71.4%)	2 (14.3%)
Methamphetamine (crystal meth, tina, crank, ice)	1 (7.1%)	0 (0%)	11 (78.6%)	2 (14.3%)
K2	1 (7.1%)	1 (7.1%)	10 (71.4%)	2 (14.3%)
Ecstasy/MDMA/Molly	(0%)	2 (14.3%)	10 (71.4%)	2 (14.3%)

Participants answered questions about their preferences of the services offered by the CCIR van. Participants were interested in accessing food (78.6%), water/beverages (78.6%), condoms and safer sex supplies (64.3%), Naloxone (64.3%), general medical care (64.3%), testing for STIs (57.1%), access to pre-exposure prophylaxis (PREP) for HIV prevention (50.0%), access to medications for substance use such as buprenorphine and naltrexone (50.0%), behavioral and mental health services (64.3%), peer support (57.1%), and needles/works (42.9%). Weekday afternoons was the most preferred time to offer services. Participants recommended specific locations for services to be offered, including “Methadone Mile,” Cambridge, Boston, Dorchester, parks, and train stations. One participant recommended “working through schools would help locate where young people tend to frequent a bit more consistently” (Table 4).

**Table 4:** Preferences for harm reduction services offered by the van reported by 14 adolescent and young adult who engage in mobile harm reduction and addiction services.

	*n*	%
What services would you be interested in accessing? (check all that apply)		
Food	11	78.6%
Water/beverages	11	78.6%
Condoms/safer sex supplies	9	64.3%
Narcan/naloxone	9	64.3%
General medical care	9	64.3%
Testing for Sexually transmitted infections	8	57.1%
Access to PREP (pre-exposure prophylaxis) for HIV prevention	7	50.0%
Access to buprenorphine (Suboxone/Subutex), naltrexone (Vivitrol), or other medications for substance use	7	50.0%
Behavioral/mental health services	9	64.3%
Peer support	8	57.1%
Needles/works	6	42.9%
Who should be on the van?		
A peer coach-someone close to my age, who has gone through some of the things I have gone through	10	71.4%
Medical provider, such as a doctor, nurse practitioner, or physician’s assistant	9	64.3%
Nurse	7	50.0%
HIV/STI counselor	6	42.9%
The age of the person/people on the van is important		
Yes	4	28.6%
No	9	64.3%
Do not know or do not want to respond	1	7.1%
Please select any language, besides English, that it would be important for people on the van to speak (check all that apply)		
Haitian Creole	6	42.9%
Spanish	8	57.1%
Cape Verdean Creole	5	35.7%
Portuguese	5	42.3%
Vietnamese	4	28.6%
Arabic	2	14.3%
Other	2	14.3%
Would you be interested in delivery of harm reduction materials by the van?		
Yes	5	35.7%
No	7	50.0%
Do not know or do not want to respond	2	14.3%
How would you prefer to communicate with the van? [check all that apply]		
Phone (call, text)	6	42.9%
Instagram	4	28.6%
Facebook	1	7.1%
Email	1	7.1%
Other	2	14.3%

Participants recommended the van be staffed with peer coaches (85.7%) and medical providers (64.3%), as well as HIV/STI counselors (42.9%). Most participants felt that the gender and age of van staff was not important (71.4%). The most important languages to offer were noted to be Haitian Creole (42.9%) and Spanish (57.1%). Five participants (35.7%) were interested in delivery of harm reduction materials by the van. They endorsed phone call and text (42.9%) and Instagram (28.6%) as the preferred means of communicating with the van for delivery (Table 4).

### 4.4 AYA and provider interview findings

We conducted five interviews with AYA who use the CCIR van services. The participants ranged from 20 to 24 years old. Three identified as male, one as female, and one as non-binary. Three participants were housed, and the other two were homeless. Two participants identified as LGBTQ+. One participant identified as Black, one as Caucasian, and the others did not specify a race during the interview recording. In addition to these participants, three providers who work on the CCIR van were interviewed.

#### 4.4.1 Facilitators to accessing community care in reach mobile harm reduction van

##### 4.4.1.1 Participant interviews

Throughout the interviews with participants, one of the most prominent themes was appreciation for the caring staff: “I just feel you guys care more, because you understand more. You guys have more experience with it than other vans have.” That participant also stated feeling that the van was “much more friendly” and “much less systematic institutionalized.” Another participant described having a case management relationship with a particular staff member, stating they connected with them “somewhat personally, like, trust and everything.” Finally, another participant felt that the staff members were helpful, stating “Everybody tries to help and everybody really-- I feel like you guys do what you need to do.” This sentiment of genuine care for the community was echoed by participant 5: “I think that was such a pivotal point in my life to just see that in action, oh here are people that are actually trying to do something within the community, and they care about doing these things, and actually trying to make it more accessible. That was really inspiring to me.”

Participants emphasized the need for non-judgmental staff. One participant described wanting “something where you can be yourself, but also still quickly get the resources that you need and also safely understand how to do harm reduction and still enjoy yourself.” Additionally, one participant described feeling uncomfortable at other harm reduction services that serve all ages: “[the syringe exchange] wasn’t what I expected. Everybody was rude, pushing,” and this participant stated that they would not feel comfortable accessing a syringe exchange or other harm reduction center with their friends.

Another characteristic of the CCIR van that participants appreciated was the wide availability of harm reduction services, “They do the fentanyl test strips and the Narcan. I feel like they are the only organization that really does it. I have not really heard any other place doing it.” Participants also described accessing safer sex supplies like condoms, HIV and hepatitis testing, “needles,” fentanyl test strips, meals, physician visits, and case management. One participant stated their appreciation for the versatility of services on the van: “You just get a new phone, sign up for housing, and get a checkup all in one day.”

##### 4.4.1.2 Provider interviews

The interviewed providers described the value they find in their role: “getting to be really patient-oriented about helping people move toward any positive change for their health, whatever that might be, and then being flexible and adapting to each patient’s stated goals.” Provider 3 shared this sentiment as well: “harm reduction…it’s a verb. It’s like this very active form of social justice where I’m going to meet people where they are at without judgment, without stigma, and I am going to help them take back their autonomy and offer them what they need.” Providers all described AYA substance use as unique from adult substance use and noted that they take a different approach when engaging AYA in harm reduction services. For example, provider 2 noted that they work to consider social networks and relationships more when working with youth. Provider 3 noted that they enjoyed the opportunity for education about harm reduction. Provider 1 emphasized the importance of considering all substances that youth may use rather than focusing exclusively on opioid use, as many young people have “experimented with” numerous substances.

Providers are hoping to address some of the barriers below through social media engagement with AYA through the roaming van model, which allows AYA to message CCIR on Instagram and get supplies delivered directly to them. Provider 1 noted that AYA participants are able to get phones through the CCIR van case management services, which may help facilitate social media or text engagement to schedule appointments in the future. This service is unique to the CCIR program: “I do not know other programs that run in the city and so I think we are hoping that this creates really easy access, low barrier access to harm reduction services.” Additionally, provider 2 noted that the van staff also conduct foot outreach to engage AYA who have not connected with them directly.

#### 4.4.2 Barriers to accessing the community care in reach mobile harm reduction van

**Participant interviews:** Participants described several barriers to accessing the CCIR mobile harm reduction van services, such as limited van hours. One participant stated, “I wish you guys was here more often,” and another said, “I think they need more capacity for people. I know so many people who need these kind of services.” Participant 5 noted that they wished the van had more space. This barrier was noted by van providers as well: “[on the] Mobile Health van, we can only fit like one or two youth at a time.” Another logistical barrier that participant 5 noted was that some youth do not use Instagram, which is how the van primarily advertises its services.

A major barrier to accessing a full range of services with CCIR was the perception of participants that their substance use was not risky. Several participants used cannabis, for example, and felt that their use was not risky: “Marijuana is from the earth, it’s natural. It’s good for you. It cures epilepsy, starvation, insomnia, cancer, it helps.” Some participants described limited understanding of harm reduction or feeling like the van services did not increase their understanding of harm reduction services. For example, when participant 4 was asked what the van taught them about substance use and harm reduction, they responded: “To be completely honest, nothing that I already did not know.” Participants 3 and 4 emphasized abstinence and reduction of use when asked about harm reduction.

Finally, as described elsewhere in this report, the COVID-19 pandemic served as a large barrier to accessing patients through the mobile harm reduction services of the CCIR van. Participant 1 said instead of seeking out services, they “just waited for, like, the outreach workers to come out if they did come out,” explicitly stating that it was “harder” to access services during the pandemic. Similarly, participant 5 reported, “Harm reduction requires for you to have this community aspect and have this face-to-face type of thing, and COVID does not allow for that. I think it makes it that much harder to reach out to people.”

**Provider interviews:** Providers noted that many young people may have a limited understanding of harm reduction: “Most people have heard of harm reduction relating to unused syringes. And so they hear harm reduction, they are like, ‘Well, that’s not me because I do not inject drugs.’” They gave another example that many AYA do not know what naloxone is when they are offered it. This limited understanding and focus on abstinence may serve as a barrier to accessing a full range of harm reduction services. In order to address this misconception, this provider states, “But I always remind people harm reduction is also like seatbelts. If you are going to drive a car, wear a seatbelt.”

Another barrier that was described by providers was difficulty engaging with youth who are at a high risk for overdose, particularly opioid overdose: “[I]t has been difficult to figure out where, like how to engage like really vulnerable youth.” Similarly, provider 3 noted that social media engagement helped connect CCIR to college students rather than more vulnerable populations. However, they still emphasized the importance of reaching this population: “I still feel really passionate about offering these services because people are often in the very early stages of their substance use, it’s usually experimental…We’re kind of more of the front lines of prevention, education.” Another provider hypothesized that some of the difficulty engaging AYA at high risk of opioid overdose may come from difficulty accessing permission to park the van in certain places: “We think there are other hot spots that we are trying to target them right now with the roaming model, but we really were not able to before because of like the politics, I guess of having the van be seen in certain communities so that felt—that was definitely a challenge that like the treatment still carries such a stigma.” Provider 2 noted that CCIR is currently working to partner with organizations that engage high-risk AYA to address this barrier. A final barrier that provider 1 reported was that during the pandemic, the common areas to use substances also shifted and CCIR van staff lost some relationships with patients, which has made it difficult to know where to engage AYA.

## 5 Discussion

We gathered varied perspectives on the CCIR van AYA pilot through chart reviews, surveys, and interviews with AYA and service providers. AYA participants largely felt that they benefited from the CCIR AYA van program and were interested in accessing a wide range of services from the AYA van in the future. Providers similarly found the van to be helpful in addressing existing gaps in harm reduction care for AYA.

Several AYA participants noted the importance of youth-designated spaces because they did not feel comfortable accessing services which catered to all ages, such as traditional needle exchange sites. AYA demonstrated different patterns of substance use and engagement with services, consistent with prior research (7, 10), requiring a program approach tailored to their preferences and patterns (8). For example, there were very few AYA with reported or documented opioid use. Many existing harm reduction services are oriented to opioid use and therefore are not useful or relevant to AYA who do not use opioids. Providers also noted that substance use among AYA is often experimental. Thus, examining harm reduction services in the context of AYA demands a wider view of harm reduction that focuses on topics more relevant to AYA, such as tobacco, alcohol and cannabis use.

Furthermore, AYA demonstrated a limited understanding of harm reduction, with many participants interpreting “harm reduction” to mean abstinence from substances. Many AYA had low perceived risk of substance use, which could limit engagement to the full range of harm reduction services available, as participants do not believe they may benefit. In particular, tobacco, alcohol, and marijuana were not viewed as needing treatment or harm reduction. Harm reduction education may lead to further reduction in risky substance use and health behaviors and aid in engagement in services.

In light of the differing needs of AYA participants, the importance of offering varied services beyond traditional harm reduction supplies was evident. Primary and urgent care services were among the most used services and should continue to be offered. Interestingly, most participants were connected to primary care and mental health services elsewhere. Nearly all patients with formal CCIR clinical encounters had prior and subsequent visits with BHCHP Family Team providers, suggesting that the CCIR van serves as an extension of Family Team services and supporting prior research that mobile services can bridge patients between office-based settings for ongoing care (8). STI testing is an essential service to offer to AYA, as this was the most common reason for clinical encounters on the CCIR van. Participants appreciated harm reduction services including distribution of safer sex supplies, Naloxone, and fentanyl test strips as well as case management services and distribution of basic essentials such as phones, food, and beverages.

In stating preferences for van services, participants noted that having caring, competent, non-judgmental providers was more important than providers of specific gender or age parameters. They appreciated providers who have experience working with AYA and with people struggling with housing instability and substance use. Providers also noted that having a social justice orientation made a difference in caring for AYA, also allowing the work to be more gratifying. Participants noted preferences for CCIR van services to be provided during weekday afternoons and evenings. They noted a wide geographic range for service provision and supply delivery, including Boston, Cambridge, Dorchester, and public settings including parks and train stations. Inability to access locations such as parks where AYA often gather due to visibility politics and lack of permission were cited by providers as a barrier to successful outreach. Discussions with local governments, organizations, and businesses about the CCIR van mission may help to expand the reach of van services.

Both providers and participants both cited the van’s capacity to deliver of services and supplies as useful in reducing barriers to access including geographic inconvenience and stigma (12, 13). Particularly in the era of COVID-19, during which interview participants reported waiting for outreach services to deliver needed services rather than seeking out services, mobile harm reduction provided needed care when AYA were unable to seek it out. Providers reported losing touch with AYA during COVID due to the pause in services. The pandemic changed where and how AYA congregate, and providers accordingly reported challenges finding and conducting outreach with AYA. Participants reported street outreach as a desired form of engagement and advertisement of services. Regular street outreach may aid in building relationships and trust among AYA as well as updating information on where AYA gather. Finally, participants endorsed social media as a desired means of advertising van services and preferred way to communicate with the van. All survey respondents reported having a phone and ability to access the internet. Some interview participants also shared they had received phones from the CCIR van. Social media may also assist in building relationships that were severed during COVID-19 and reduce access barriers by coordinating delivery of supplies and services where they are most needed.

Summary of recommendations for AYA harm reduction programs.

**Table:** 

Goal	Recommendations
Focus harm reduction efforts to AYA’s unique needs and perceptions	Offer harm reduction education for substances more relevant to AYA (tobacco, alcohol, cannabis) since these substances are perceived as “low-risk” Staff van with providers who have experience working with AYA Other desired services: Primary and urgent care services STI testing Harm reduction supplies: safer sex kits, Naloxone, fentanyl test strips Case management Basic essentials: phones, food, and beverages
Reduce barriers to access	Preferred van hours include weekday afternoons and evenings Wide geographic range: Boston, Cambridge, Dorchester Gain access public settings where AYA often gather such as parks, train stations, skate parks, by engaging with local governments and businesses
Explore new ways to engage AYA such as street outreach, delivery services, and social media	Offer street outreach as a form of engagement and advertising of services: regular street outreach may aid in building relationships and trust among AYA as well as updated information on where AYA gather Offer delivery services: mobile harm reduction can provide needed care when AYA are unable to seek it out Social media can be used to advertise van services or communicate with AYA for coordination of supply delivery

### 5.1 Conceptual constraints

With the resumption of services, the volume of AYA encountered on the van was much lower than expected, despite the re-orientation of services. We set out to conduct a robust mixed methods evaluation including surveys of AYA who use CCIR services and interviews of key stakeholders including patients, providers, and local members of the Downtown Business Improvement District (BID). Unfortunately, due to the lower volume of patients on the van, we encountered significant challenges in completing the numbers of surveys and interviews with AYA despite persistent outreach to AYA during van hours and through social media outreach. None of the BID members contacted agreed to an interview despite phone, email, and in-person outreach to twenty businesses. The small sample size of survey and interview participants makes it challenging to generalize results widely, though there are valuable lessons to be learned in their responses. Additionally, we employed chart review to understand the nature of clinical visits on the van, however this method is limited by the amount of information that was documented by providers. Often, supplies such as safe use kits and naloxone are frequently provided by van workers and not documented by clinical providers, thus we were unable to accurately report provision of these harm reduction supplies based on chart review alone. Our surveys are impacted by the interpretation of the participants. For example, while only one participant reported injection drug use in the past year, two participants reported obtaining unused needles and works from a needle exchange/drop-in center; this apparent discrepancy could represent that a participant collected supplies for someone else, collected supplies prior to the last year, obtained other non-injection related supplies, or mis-interpreted the question.

## 6 Conclusion

The CCIR van AYA Pilot Program demonstrates its ability to provide high quality care and harm reduction services to AYA while reducing previously noted barriers to harm reduction for AYA, including lack of youth-oriented services, geographic inconvenience, and lacking caring, knowledgeable providers. Our evaluation also demonstrates opportunities to expand the reach and impact of the AYA program, including increased capacity and hours, greater diversity of outreach sites and locations, AYA oriented harm reduction education, and continued street and social media outreach. Focusing on these aspects may help the CCIR AYA program excel in offering relevant services and constantly evolving with the changing needs of the AYA community.

## Data availability statement

The original contributions presented in the study are included in the article/Supplementary material, further inquiries can be directed to the corresponding author.

## Ethics statement

The studies involving humans were approved by Boston Medical Center and Boston University Medical Campus Institutional Review Board. The studies were conducted in accordance with the local legislation and institutional requirements. The ethics committee/institutional review board waived the requirement of written informed consent for participation from the participants or the participants’ legal guardians/next of kin because verbal consent was obtained during the interviews.

## Author contributions

EY: Data curation, Writing – original draft, Writing – review & editing. EH: Data curation, Formal analysis, Writing – original draft, Writing – review & editing. EN: Conceptualization, Data curation, Writing – original draft, Writing – review & editing. AC: Conceptualization, Data curation, Formal analysis, Funding acquisition, Methodology, Project administration, Supervision, Writing – original draft, Writing – review & editing.
