# Supplementary figures and images for: Evaluating mobile harm reduction services for youth and young adults
Source: Front Public Health. 2024 May 22;12:1375323. doi: 10.3389/fpubh.2024.1375323 (PMC11150819; doi:10.3389/fpubh.2024.1375323)

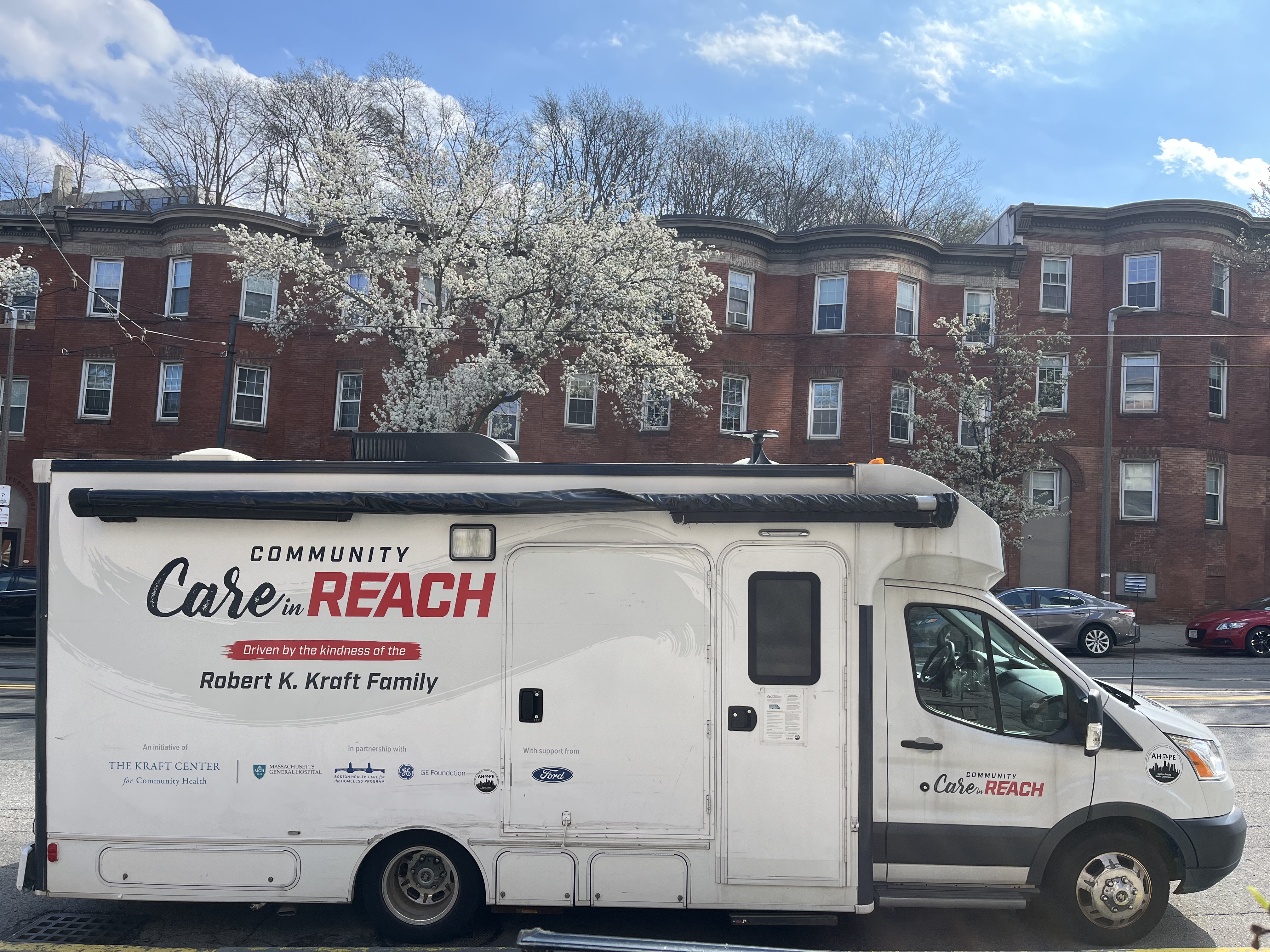

Supplement: Supplementary file 1 [file Image_1.jpeg]

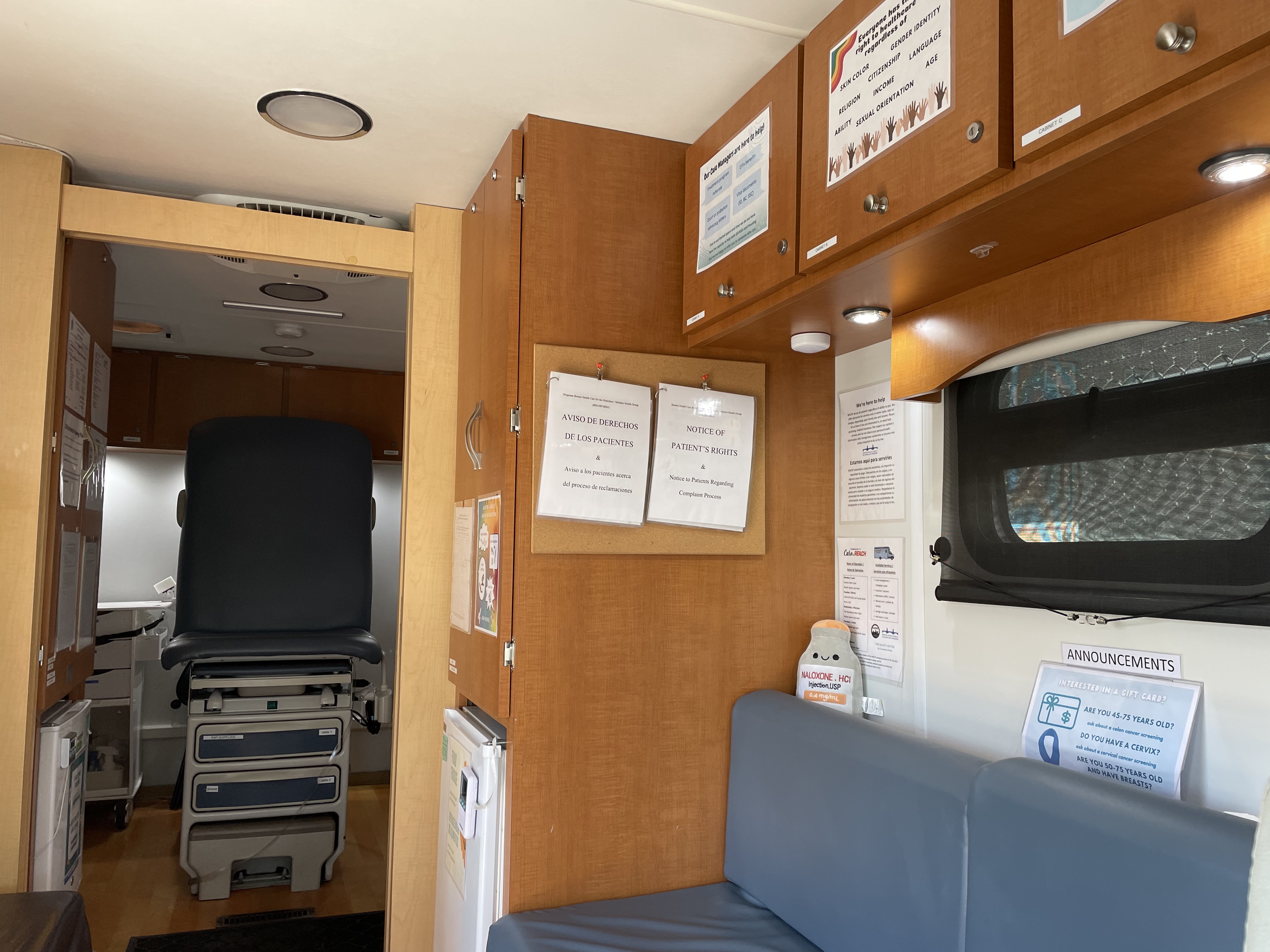

Supplement: Supplementary file 2 [file Image_2.jpeg]

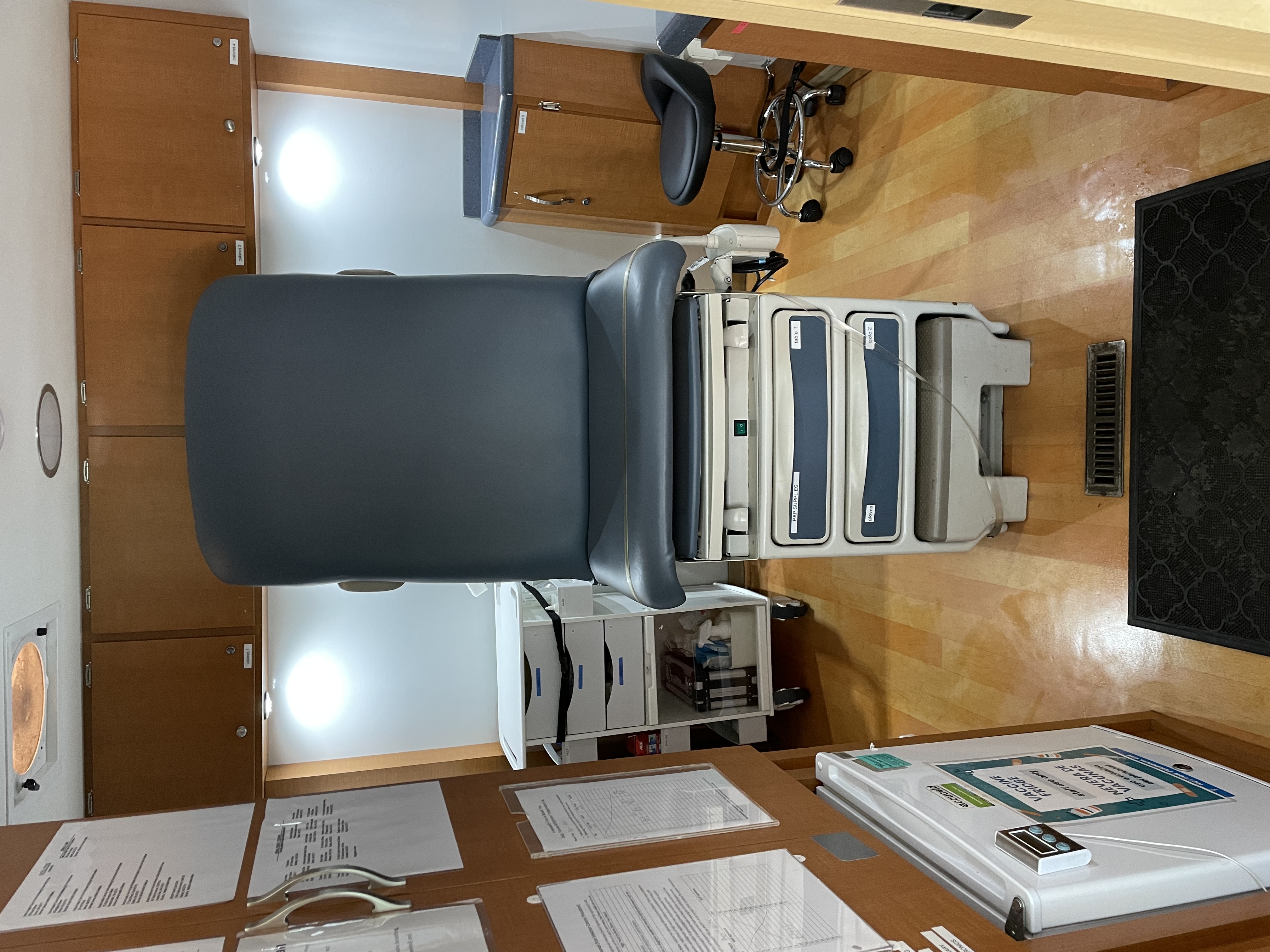

Supplement: Supplementary file 3 [file Image_3.jpeg]
